# Detection of Gene Expression in an Individual Cell Type within a Cell Mixture Using Microarray Analysis

**DOI:** 10.1371/journal.pone.0004427

**Published:** 2009-02-12

**Authors:** Penelope A. Bryant, Gordon K. Smyth, Roy Robins-Browne, Nigel Curtis

**Affiliations:** 1 Department of Paediatrics, The University of Melbourne, Royal Children's Hospital Melbourne, Parkville, Victoria, Australia; 2 Microbiology & Infectious Diseases Research Group, Murdoch Children's Research Institute, Royal Children's Hospital Melbourne, Parkville, Victoria, Australia; 3 Infectious Diseases Unit, Department of General Medicine, Royal Children's Hospital Melbourne, Parkville, Victoria, Australia; 4 Bioinformatics Division, Walter and Eliza Hall Institute of Medical Research, Parkville, Victoria, Australia; 5 Department of Microbiology & Immunology, University of Melbourne, Parkville, Victoria, Australia; University of Calgary, Canada

## Abstract

**Background:**

A central issue in the design of microarray-based analysis of global gene expression is the choice between using cells of single type and a mixture of cells. This study quantified the proportion of lipopolysaccharide (LPS) induced differentially expressed monocyte genes that could be measured in peripheral blood mononuclear cells (PBMC), and determined the extent to which gene expression in the non-monocyte cell fraction diluted or obscured fold changes that could be detected in the cell mixture.

**Methodology/Principal Findings:**

Human PBMC were stimulated with LPS, and monocytes were then isolated by positive (Mono+) or negative (Mono−) selection. The non-monocyte cell fraction (MonoD) remaining after positive selection of monocytes was used to determine the effect of non-monocyte cells on overall expression. RNA from LPS-stimulated PBMC, Mono+, Mono− and MonoD samples was co-hybridised with unstimulated RNA for each cell type on oligonucleotide microarrays. There was a positive correlation in gene expression between PBMC and both Mono+ (0.77) and Mono− (0.61–0.67) samples. Analysis of individual genes that were differentially expressed in Mono+ and Mono− samples showed that the ability to detect expression of some genes was similar when analysing PBMC, but for others, differential expression was either not detected or changed in the opposite direction. As a result of the dilutional or obscuring effect of gene expression in non-monocyte cells, overall about half of the statistically significant LPS-induced changes in gene expression in monocytes were not detected in PBMC. However, 97% of genes with a four fold or greater change in expression in monocytes after LPS stimulation, and almost all (96–100%) of the top 100 most differentially expressed monocyte genes were detected in PBMC.

**Conclusions/Significance:**

The effect of non-responding cells in a mixture dilutes or obscures the detection of subtle changes in gene expression in an individual cell type. However, for studies in which only the most highly differentially expressed genes are of interest, separating and analysing individual cell types may be unnecessary.

## Introduction

The choice between using cells of a single type and a mixture of cells in microarray-based analysis of global gene expression is difficult. Analysing cells of one type, such as monocytes[1], [2], [3], necessitates an isolation step that may be technically difficult[4] and which may induce non-specific changes in gene expression. Analysing a cell mixture, such as peripheral blood mononuclear cells (PBMC)[5], [6], [7], is simpler but gene expression changes in cells of a specific cell type, particularly if present in small numbers, may not be detected. Specifically, significant changes of gene expression in the cell type of interest may be undetectable as a result of the dilutional effect of a majority of non-responding cells or obscured by opposite responses in other cell types. In addition, it is impossible to attribute observed differential expression to individual cell types.

This study compared gene expression in PBMC with expression in monocytes alone after stimulation with lipopolysaccharide (LPS) as a paradigm for determining the extent to which changes in gene expression in a particular cell type can be detected in a cell mixture. A strategy was used that preserved the advantage of the cellular interactions of PBMC but which allowed detection of gene expression from an individual cell type by separating monocytes *after* stimulation of PBMC *ex vivo*. Analysis was undertaken in monocytes isolated by both positive and negative selection to control for possible gene expression induced by CD14-mediated pathways. Gene expression was also measured in the non-monocyte cell fraction remaining after positive selection to provide information about the effect on gene expression in PBMC. The proportion of LPS-induced differentially expressed monocyte genes that can be detected in PBMC was successfully quantified.

## Results

Global gene expression in LPS-stimulated PBMC from two individuals was compared to expression in monocytes isolated from the same blood sample. Monocytes separated by two different methods – positive isolation (Mono+) and negative isolation (Mono−) – were compared to PBMC. Duplicate PBMC samples (PBMC1 and PBMC2) were stimulated in parallel for 3 and 24 hours to estimate the variability between independent stimulations of the same cell population. The PBMC and monocyte samples were compared to the non-monocyte cell fraction remaining after positive isolation (MonoD), comprising PBMC depleted of Mono+ cells, to analyse the effect of the non-monocyte cells in the mixture on gene expression.

The proportion of monocytes in the PBMC samples was 10–14% (table 1). This proportion was greater after both positive and negative monocyte isolation. The monocyte component of Mono+ could not be measured directly, but was implied to be 100%, as the MonoD sample contained 0% CD14-positive cells.

**Table 1:** Percentage of monocytes in different samples.

	CD14+ cells (%)	
	Subject 1	Subject 2
**Mono+**	100	100
**Mono−**	70.8	85.0
**PBMC**	14.2	10.5
**MonoD**	0.1	0.0

RNA from each stimulation time point was competitively hybridised with RNA from 0 hours of the same cell type (eg Mono+ at 3 h with Mono+ at 0 h). The 0 hour sample was chosen as the reference as it was biologically relevant and could be used to compare changes between cell types.

### Overall patterns of expression

**Figure 1:**
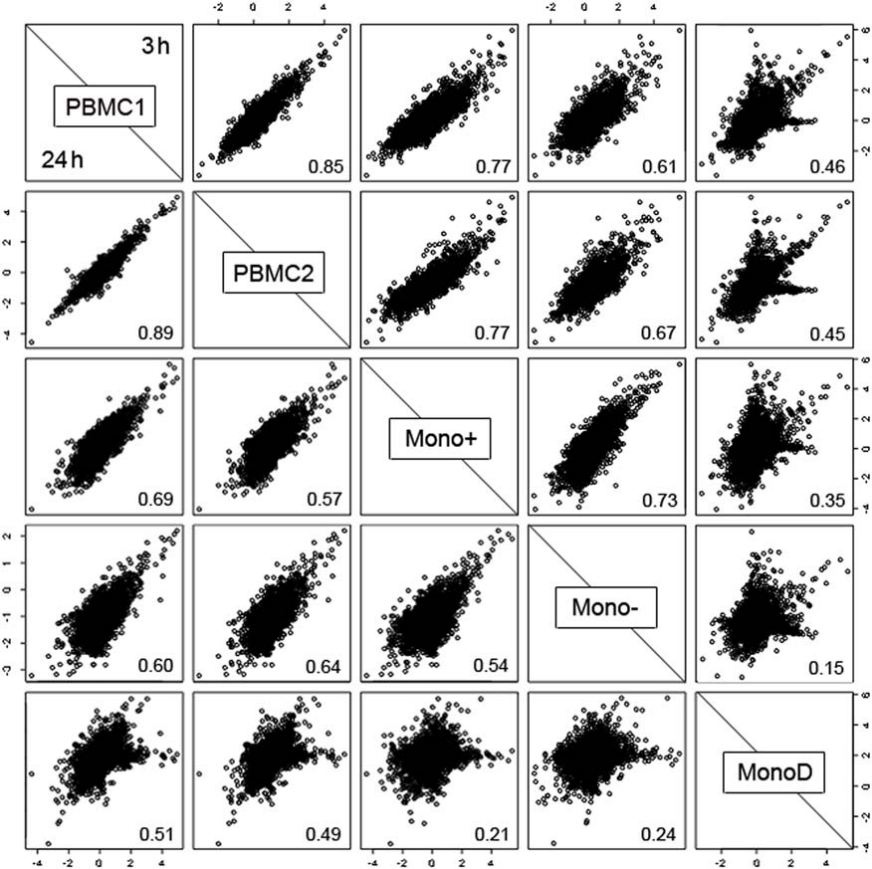
Matrix of scatter plots showing estimated log2-fold changes for each cell population. The log-fold changes for each sample are plotted against those for each of the other samples for gene expression after stimulation with LPS for 3 hours (top right of diagonal) and 24 hours (bottom left). Correlation coefficients between each pair of samples are shown in the bottom right of each box.

The broad relationship between gene expression in the different samples was illustrated by plotting the log ratios of differential expression between LPS-stimulated and unstimulated samples for the individual for whom there were repeat PBMC samples. There was a positive correlation between overall gene expression detected between all the samples containing monocytes after both 3 and 24 hours' stimulation with LPS (figure 1). The pattern of gene expression was most similar between the two PBMC samples with a correlation coefficient of 0.85 (3 hours) and 0.89 (24 hours). There was also a positive correlation between both of the PBMC samples and the monocyte samples, with the Mono+ sample having greater correlation at 3 hours (0.77) than the Mono– sample (0.61–0.67). There was a low correlation at both time points (0.15–0.35) between both the Mono+ and Mono− samples and the MonoD samples as expected. The fold changes in gene expression between unstimulated and LPS-stimulated samples tended to be lower in the PBMC than in the monocytes, consistent with the dilutional effect of non-monocyte cell expression in PBMC.

**Figure 2:**
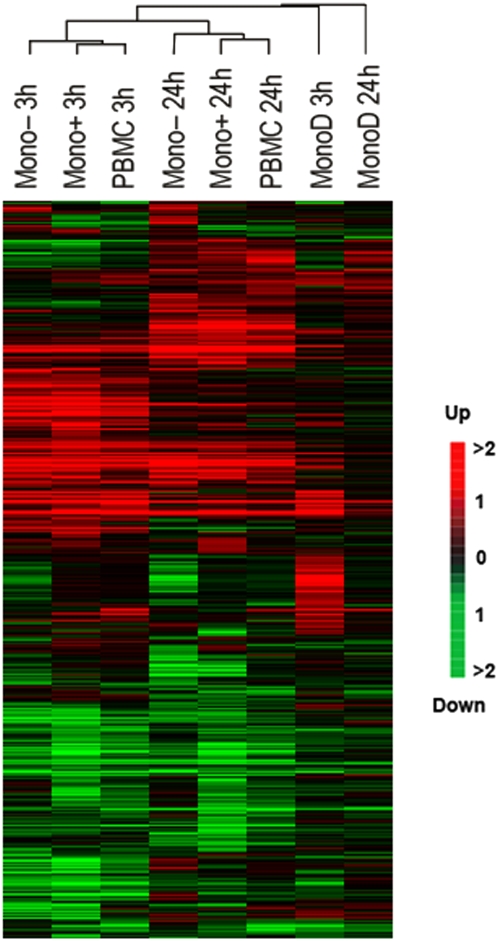
Hierarchical cluster analysis using average linkage of the linear model estimated log-expression of all genes and cell types at 3 and 24 hours.

Hierarchical clustering analysis of gene expression after LPS stimulation at both time points confirmed that the two PBMC samples clustered together most closely at each time point, followed by the Mono+ and Mono− samples (figure 2). The 3 hour samples and the 24 hour samples clustered together respectively, indicating that there was more similarity at each time point between gene expression in the different samples that contained monocytes, than there was between the same sample types at different times. The PBMC and monocyte samples from both time points clustered together more closely than either MonoD sample, indicating these samples are the most dissimilar.

### Differentially expressed genes

A linear model was fit to the data from both individuals to compare differential gene expression induced by LPS in monocytes and PBMC. The Mono+ samples were used as the ‘gold standard’as they comprised the purest monocyte populations. The MonoD samples were used to provide information about the effect of the non-monocyte cell fraction on gene expression in PBMC.

**Figure 3:**
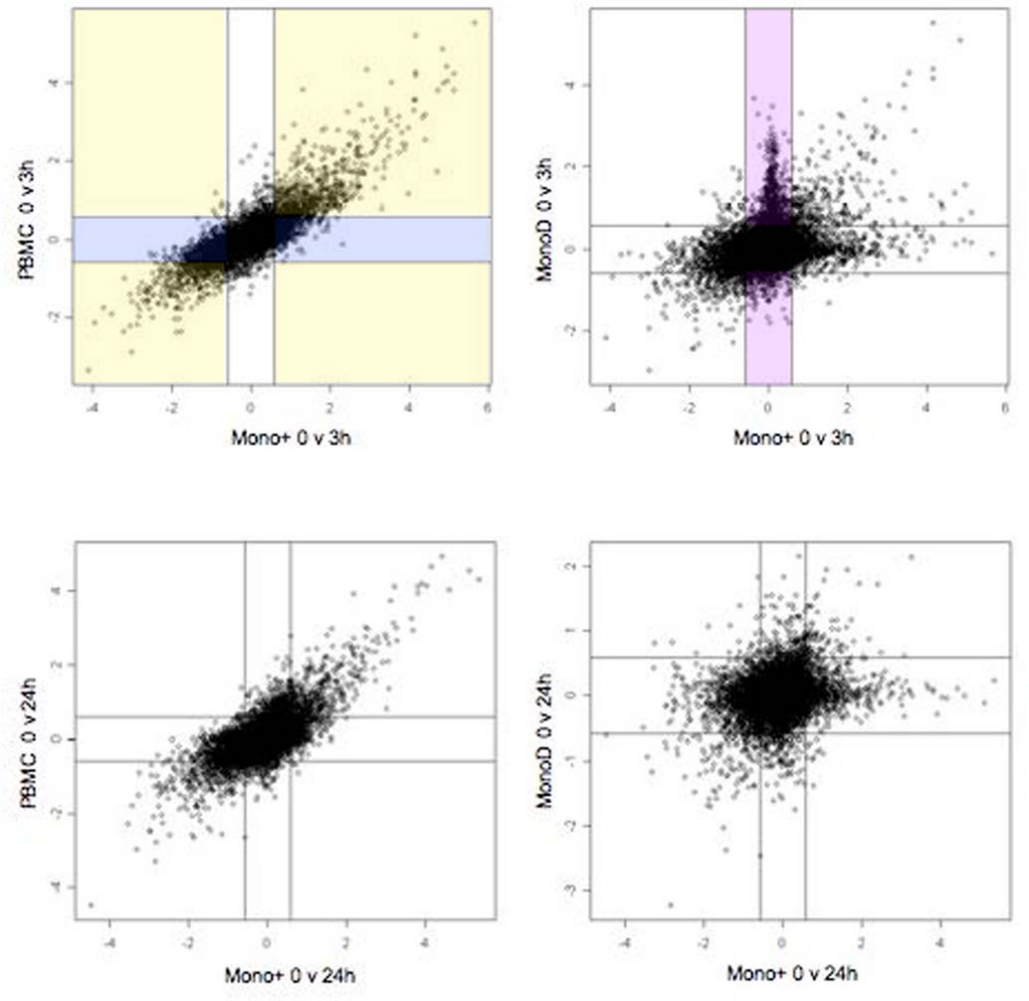
Scatterplots showing log2-fold changes for monocytes (Mono+) compared with non-monocytes (MonoD) and PBMC at 3 and 24 h. Each point corresponds to a gene and represents its differential expression after stimulation with LPS for 3 or 24 h compared to 0 h. The lines represent the cut-off of 50% fold change. Each of the 9 regions separated by the cut-off lines per graph corresponds to a cell in Table 2.

After 3 hours' stimulation with LPS, 1716 genes were significantly differentially expressed in the Mono+ samples (872 up, 844 down), underlining the marked effect of LPS on monocytes (table 2, figure 3). Of these 1716 genes, only 791(46%, shaded yellow) were detected as differentially expressed in PBMC after stimulation with LPS, and 3 of these genes (0.2%) were expressed in the opposite direction to the Mono+ sample. The dilutional effect of the lack of gene expression in non-monocytes on the differential expression detected in PBMC was explored. This was done by examining the proportion of genes that did not change in response to LPS in the MonoD sample. Of the 1716 genes that were differentially expressed in the Mono+ sample, most (87%, shaded violet) did not change in the MonoD sample. The influence of gene expression in non-monocytes in the opposite direction to that in monocytes was also explored, and found to occur in only a small minority (0.8%) of the differentially expressed genes in the Mono+ sample. A further 361 genes (shaded blue) were differentially expressed after LPS stimulation in PBMC but not in the Mono+ sample, reflecting changes in expression in the non-monocyte cells.

**Table 2:** Comparison of change in expression between Mono+ and MonoD and PBMC samples at 3 and 24 hours (each cell corresponds to a region in Figure 3).

Mono+	Time point	MonoD			PBMC		
		Down	No change	Up	Down	No change	Up
Down	3 h	243	1382	43	433	1227	8
No change		214	14519	881	87	15381	146
Up		19	1086	277	0	736	646
Down	24 h	114	1384	23	390	1125	6
No change		151	15895	156	171	15533	498
Up		9	857	75	4	495	442

The findings were similar at 24 hours: 1453 genes were significantly differentially expressed in response to LPS in the Mono+ sample (695 up and 758 down). Of these 1453 monocyte genes, only 593 (41%) were detected as being differentially expressed in PBMC. A further 471 genes were differentially expressed after LPS stimulation in PBMC but not in the Mono+ sample. Again, both of these findings were due to the mainly dilutional effects of expression in the non-monocytes: of the 1453 monocyte genes, 94% were not differentially expressed in the non-monocytes in response to LPS while 0.3% changed in the opposite direction.

**Figure 4:**
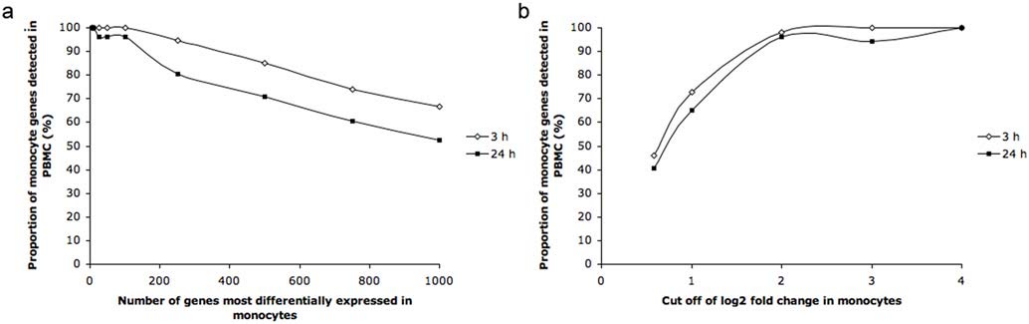
The proportion of differentially expressed genes in PBMC in relation to (a) the number selected of top ranked differentially expressed genes in the monocyte population, and (b) the cut-off selected for fold change in monocyte genes after LPS stimulation.

To determine the effect on detection in PBMC in the most highly differentially expressed monocyte genes, genes were ranked by fold change in the Mono+ sample and the most differentially expressed genes were selected. Detection of these genes in PBMC was investigated by selecting different numbers of the topmost differentially expressed genes: from the top 10 genes to the top 1000 genes (figure 4a). As an example, all of the 100 monocyte genes most differentially expressed in response to LPS were detected in PBMC at 3 hours, and 96 of the top 100 monocyte genes at 24 hours. The proportion of genes detected in PBMC decreased with increasing numbers of genes selected, confirming that magnitude of expression in monocytes affected detection in PBMC. This is also illustrated by relating the proportion of LPS-induced gene expression changes in monocytes detectable in PBMC to their fold change (figure 4b).

### Effects on individual gene expression

The expression of a number of specific individual genes involved in the immune response to LPS was investigated further to compare expression in different cell types.

**Figure 5:**
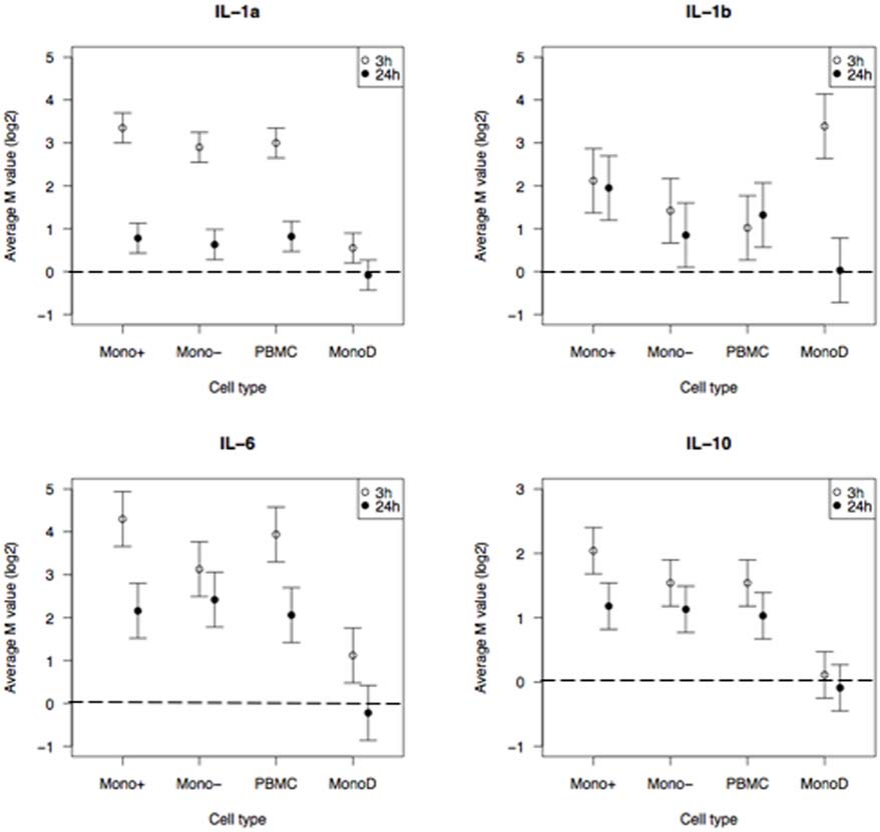
Comparison of IL-1a, IL-1b, IL-6 and IL-10 gene expression in different samples after stimulation with LPS for 3 and 24 hours. The log2-fold change (M value) and standard error for the expression of each gene are plotted for each sample.

First, the expression of ‘validation’ genes encoding cytokines likely to be differentially expressed in monocytes in response to LPS on the basis of previous published data was investigated[8], [9], [10]. The expression of interleukin (IL)-1a, IL-1b, IL-6 and IL-10 genes was upregulated as expected, with expression after 3 hours' LPS stimulation being more upregulated than after 24 hours (figure 5). There was concordance in expression for these differentially expressed genes in monocytes and PBMC samples. TNF expression was increased at 3 hours as expected and, although statistically significant, did not quite reach the cut-off for fold change. This likely reflects the fact that TNF expression induced by LPS is known to peak earlier than 3 hours[11].

**Figure 6:**
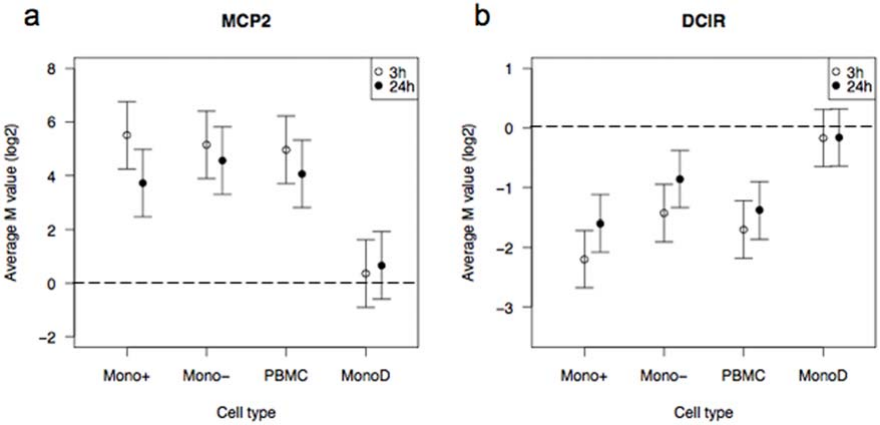
Genes expressed in common between monocytes and PBMC. Examples of gene expression that is (a) upregulated and (b) downregulated in both PBMC and monocyte samples after stimulation with LPS for 3 and 24 hours. The log2-fold change (M value) and standard error for the expression of each gene are plotted for each sample.

Likewise, there were other inflammatory response genes differentially expressed after LPS stimulation that were detected in both PBMC and monocytes. These included genes that were upregulated, such as monocyte chemotactic protein 2 (MCP2), and genes that were downregulated, such as dendritic cell immunoreceptor (DICR), confirming that genes strongly differentially expressed in monocytes can be detected in PBMC (figure 6).

**Figure 7:**
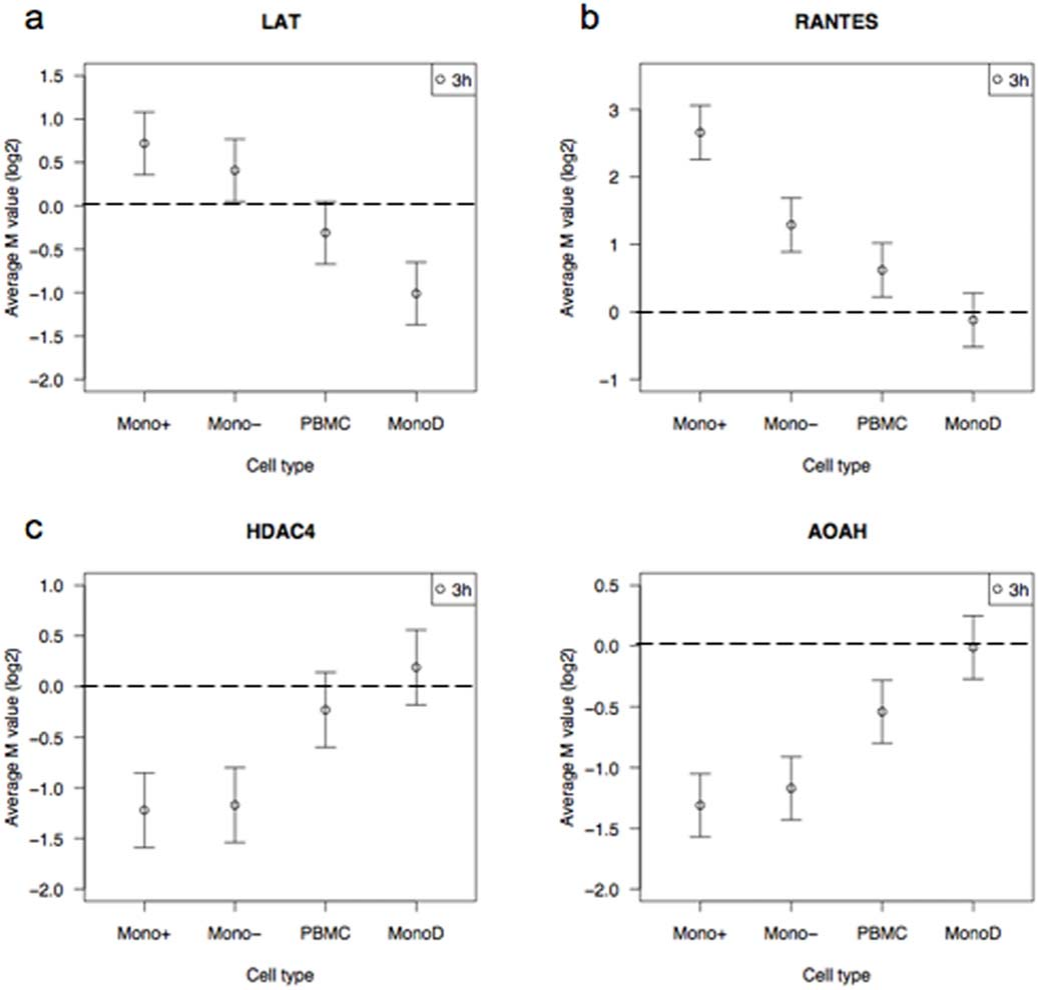
Genes expressed differentially between monocytes and PBMC. Examples of gene expression that is (a) upregulated in monocytes but not PBMC, (b) upregulated in monocytes and remains detectable in PBMC and (c) downregulated in monocytes but not PBMC after stimulation with LPS for 3 hours. The log2-fold change (M value) and standard error for the expression of each gene are plotted for each sample.

Of particular interest were genes that had significant differential expression after LPS stimulation in monocytes that were not detected when PBMC were used for analysis (figure 7). The linker for activation of T cells (LAT) gene, known to be upregulated in monocytes[12], was significantly upregulated at 3 hours in the Mono+ and Mono− samples. Conversely, expression of this gene was significantly downregulated in the MonoD sample, resulting in an overall lack of change in expression in the PBMC sample. There were, however, also genes where the differential expression in response to LPS appeared markedly different between monocytes and PBMC, but were detectable in both samples. For example, the small inducible cytokine A5 (RANTES)[13] was highly differentially expressed in the Mono+ sample. Although there was no differential expression in the MonoD sample, the marked expression in monocytes resulted in the gene remaining detectable in PBMC, despite the dilutional effects of the non-monocytes. There were in addition genes in which downregulation of expression in monocytes was more significant than in PBMC. These included histone deacetylase 4 (HDAC4)[14], and acyloxyacyl hydrolase (neutrophil) (AOAH)[15], where in both cases the differential expression in the MonoD sample was negligible or even slightly upregulated. These examples highlight the different effects of non-monocyte cells on overall expression in PBMC, obscuring or diluting the expression detectable in monocytes.

**Figure 8:**
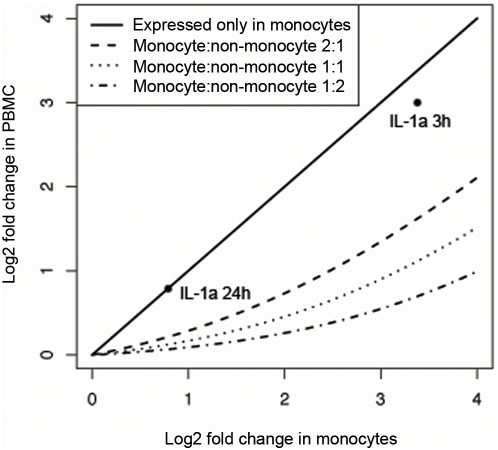
The fold change in gene expression after LPS-stimulation predicted in PBMC as a function of fold change in monocytes. The top curve is for genes expressed only in monocytes with no attenuation of fold change. The other curves assume that the gene is expressed in monocytes at double, equal and half the level that it is in non-monocytes. Expression of the IL-1a gene at 3 and 24 hours is plotted as an example.

The known proportion of monocytes (a mean of 12.4% in this study) and non-monocytes within the PBMC was used to predict gene expression in PBMC. This was done by calculating the reduction in fold change in gene expression that would occur in PBMC with different expression levels in non-monocytes (figure 8). Increased expression in non-monocytes leads to greater dilution of the fold change in PBMC. This is illustrated by expression of the IL-1a gene. At 24 hours, IL-1a is expressed only in monocytes (figure 5) and the fold change is therefore the same in monocytes and PBMC (figures 8). In contrast, at 3 hours IL-1a is expressed in non-monocytes at about one sixth of the level in monocytes and this results in a reduction in fold change in PBMC (figure 8).

**Figure 9:**
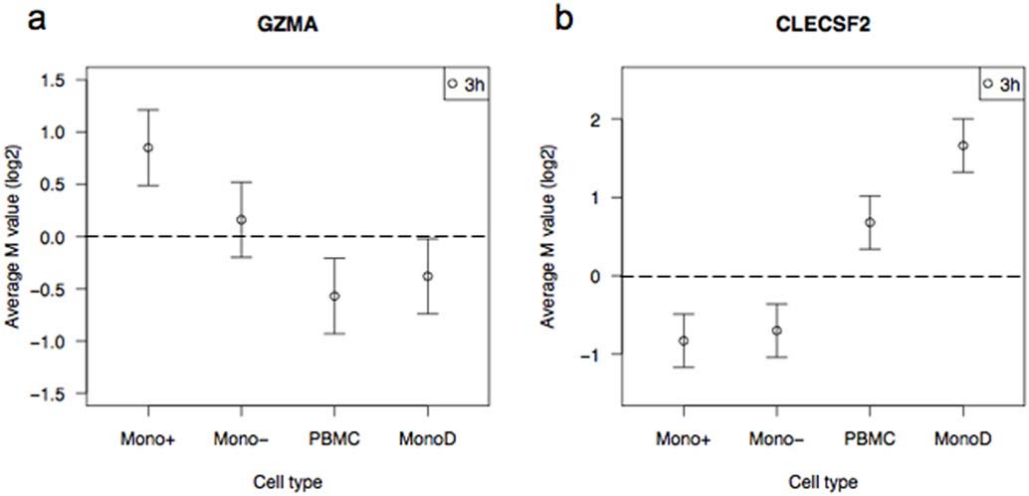
Examples of genes that are expressed in opposite directions in monocytes and PBMC after stimulation with LPS for 3 hours. The log2-fold change (M value) and standard error for the expression of each gene are plotted for each sample.

There were a few genes that were differentially expressed in opposite directions in monocytes and PBMC after stimulation with LPS. Examples included granzyme A (GZMA) and c-type lectin (CLECSF2) (figure 9). In both cases, expression in the MonoD sample was in the opposite direction to the expression in the monocyte samples, which resulted in opposite overall expression in PBMC. These are the most extreme examples of the effect of differential expression in non-monocyte cells. For these few genes, analysis of PBMC provides misleading information about expression in monocytes.

Several functional gene ontology (GO) terms were found to be highly statistically over-represented amongst the differentially expressed genes. Not surprisingly GO terms that were similarly over-represented in genes differentially expressed after LPS stimulation in both monocytes and PBMC were related to the immune response – for example: response to biotic stimulus, defense response, inflammatory response and immune response, and these include genes such as TNF and MCP2. The GO terms statistically over-represented in the genes that were expressed most differently between monocytes and PBMC included more specific immune activities – for example: regulation of T cell activation (eg LAT), chemotaxis (eg RANTES), lymphocyte differentiation (eg HDAC4) and B cell differentiation (eg HDAC5). In addition to the activity being more specified, the cell type was more specific, reinforcing the findings of different expression in different cell types.

## Discussion

In this study, we quantified the proportion of LPS-induced differentially expressed monocyte genes that could be measured in PBMC, and determined the extent to which gene expression in the non-monocyte cell fraction diluted or obscured fold changes that could be detected in the cell mixture. The effect of gene expression in the non-monocyte fraction diluting or obscuring the detection of expression of particular genes in PBMC was not insubstantial. More than half of the statistically significant LPS-induced changes in gene expression in monocytes were not detectable in the PBMC sample. However, the genes whose expression were most significantly changed in monocytes in response to LPS were those that could also be detected in PBMC as a whole. In our study, almost all genes (97%) with a greater than four fold change were still detectable (figure 4b).

Future studies to quantify in more detail the diluting effect of differential expression in non-target cells on detectable gene expression of an individual cell type within a mixture, could be undertaken by means of titration experiments[16]. A series of artificially mixed samples could be used, with, for example, decreasing proportions of MonoD added to Mono+ samples.

We chose to study the interaction between monocytes and LPS because these inflammatory cells comprise a significant minority of PBMC (in this study 10–14%) and their interaction with LPS in initiating the inflammatory cascade in Gram negative bacterial infection is well described. It is likely that our results apply to the detection of gene expression in other situations. The proportion of genes in a particular cell type that can be detected in a cell mixture might be lower for target cells comprising a smaller proportion of the mixture or in which the cell type of interest is not the principal target or primary effector cell. However, it is probable that detection of the most differentially expressed genes would remain less susceptible to the influence of other cells within the cell mixture.

To ensure gene expression induced by LPS stimulation was comparable in the monocytes analysed alone and in those analysed within PBMC, monocytes were separated after stimulation of PBMC with LPS, thus preserving the interactions between cells. The repeatability of our results was confirmed by the high degree of correlation between the two PBMC samples analysed from the same individual. This control validated the relevance of the differences in gene expression detected between PBMC and the monocyte samples.

We included a number of other control samples to exclude the possibility that our results were confounded by artefactual or technical variability. Monocytes were isolated by different methods to enable us to control for the possibility that gene expression might be induced by binding of CD14 antibodies during positive selection of monocytes. The similarity in differential gene expression between the Mono+ and Mono− samples showed that changes in cell surface expression of CD14 or LPS-binding of CD14 did not have a major effect. The greater correlation between the PBMC and Mono+ samples than between the PBMC and Mono− samples reflects the higher proportion of monocytes selected using positive isolation. Negative isolation resulted in up to 50% of non-monocytes cells remaining in the Mono− sample. Possible explanations include the relatively lower efficacy of negative selection and the absence of antibodies to every non-monocyte cell type present in PBMC.

Artefactual or technical variability as a cause of differences in gene expression between samples was also excluded by the finding that the samples that shared the most similar technical processes (Mono+ and MonoD) had the most different gene expression, whilst those that underwent the most different processes (Mono+ and Mono−) had the most similar expression.

Our study has important implications for the design and analysis of microarray-based studies. For studies in which detection of subtle changes in gene expression in a specific cell type is important, our study shows that analysing a cell mixture will miss a substantial proportion of changes. However, when only the most highly differentially expressed genes are of interest, it may not be necessary to undertake time-consuming and costly separation and analysis of individual cell types.

## Materials and Methods

This study was reviewed and approved by the Human Research Ethics Committee (23096A) at the Royal Children's Hospital, and informed consent was obtained verbally from the adult volunteers.

### PBMC separation and stimulation

Blood from two healthy volunteers was collected into tubes containing endotoxin-free lithium heparin (Becton Dickinson, Franklin Lakes, NJ, USA). PBMC were separated using a Ficoll-Paque™ gradient (Amersham Biosciences, Uppsala, Sweden). Triplicate aliquots from each individual of 2×10^6^ PBMC were simultaneously stimulated with 1 µg/ml LPS (Sigma Aldrich, Sydney, NSW, Australia) and incubated at 37°C with 5% CO_2_ for 0, 3 and 24 hours. The triplicates at each time point were treated identically to enable comparison between the PBMC samples and two monocyte samples that would subsequently be separated. TRIzol® (Invitrogen Life Technologies, Carlsbad, CA, USA) was added to all samples before storing at −80°C.

### Monocyte separation

Monocytes were separated by two different methods. Positive isolation: monocytes were positively selected by incubation for 60 min at 4°C with magnetic Dynabeads® M-450 CD14 (Dynal Biotech, Carlton South, Victoria, Australia) coated with anti-CD14 monoclonal antibody. The positively-selected monocytes were isolated from the sample with a Dynal MPC® magnet and the monocyte-depleted supernatant transferred to another tube and centrifuged. Negative isolation: monocytes were isolated by the removal of other cell types using a commercial kit (Dynal® Monocyte Negative Isolation Kit). In addition to gamma globulin to block Fc receptors on monocytes, a mixture of mouse monoclonal antibodies for CD2, CD7, CD16, CD19, CD56 and CD 235a was added to the PBMC sample. After incubating and washing the cells, the Depletion Dynabeads® coated with an Fc specific human antibody against mouse IgG were added and incubated for 30 min at 4°C. A Dynal MPC® magnet was used to isolate beads attached to the unwanted cells. The supernatant containing the negatively-isolated monocytes was removed and centrifuged. After isolation, TRIzol® was added to the three samples: positively-isolated monocytes (Mono+), the cellular fraction depleted of monocytes after positive isolation (MonoD) and the negatively-selected monocytes (Mono−), before storing at −80°C.

### Cell population analysis by flow cytometry

For each of the PBMC, Mono− and MonoD samples, 5×10^5^ cells were stained with PE-conjugated CD14 (IOTest®, Immunotech, Marseille, France) and 5×10^5^ cells were stained with PE-conjugated mouse IgG_1_ as a negative isotype control. Cells were incubated in phosphate buffered saline (PBS), 0.1% sodium azide and 20 µl of the conjugated antibody at room temperature for 15 min, washed and resuspended in 300 µl PBS with 2% formalin. Analysis was undertaken using a LSR II flow cytometer and BD FACSDiva® software (Becton Dickinson). The Mono+ samples were not measured directly although selection with anti-CD14 beads is likely to select CD14-positive cells at an efficiency approaching 100%. This contention is supported by the MonoD sample having almost 0% CD14-positive cells.

### RNA preparation

RNA was isolated by chloroform:phenol extraction[17] and purified using a kit (RNeasy™, Qiagen, Clifton Hill, Victoria, Australia) following the manufacturer's protocol. Linear amplification of RNA was performed using a kit (MessageAmp™ II aRNA, Ambion, Austin, TX, USA) following the manufacturer's protocol. RNA quality was assessed by A_260_/A_280_ ratio in addition to analysis in a Bioanalyzer 2100 (Agilent Technologies, Forest Hill, Victoria, Australia). All RNA samples were of a satisfactory quality.

### Microarray hybridisation

The study used 36 spotted microarrays printed with the Compugen human 19,000 oligonucleotide library (http://www.cgen.com) and a selection of control probes at the Adelaide Microarray Facility (Adelaide, Australia). To minimize variability, all microarrays were from the same printing batch. Amplified RNA (aRNA) was labelled by a direct platinum-based labelling technique using a kit (ULS™ aRNA labelling, Kreatech Biotechnology, Amsterdam, The Netherlands) following the manufacturer's protocol. For each sample 2 µg aRNA was labelled with 2 µl of ULS™-Cy dye. For each pair of aRNA samples to be hybridised to a slide, one sample was labelled with ULS™-Cy3 and one with ULS™-Cy5. aRNA from each stimulation time point was competitively hybridised with aRNA from 0 hours of the same cell type from the same individual. The samples from each individual were hybridised separately. The two dye-coupled samples for each array were combined and fragmented using 4 µl Fragmentation Reagents (Ambion). The labelled sample was mixed with 10 µl 1 mg/ml human Cot-1 DNA (Invitrogen), 15 µl 20×SSC, 20 µl deionised formamide (Sigma Aldrich), 20 µl Kreatech solution (Kreatech Biotechnology) and 5 µl 10% SDS, heated at 95°C for 5 min and cooled to room temperature. Each sample was applied to a slide which was incubated in a water bath in the dark at 42°C for 18 hours, washed and scanned using a Genepix® 4000B scanner (Molecular Devices, Sunnyvale, CA, USA). Every hybridisation was performed in duplicate, and the dyes were swapped in a balanced design to counteract any possible probe-specific dye effects.

### Microarray data normalisation and analysis

Each scanned TIF image was quantified using Genepix Pro 6.0 software (Molecular Devices) to obtain foreground and background intensity values for each spot. Genepix was configured to generate the custom morphological close-open background estimator, which is less variable than the more usual local background estimators[18]. All normalization and differential expression analysis was conducted using the limma software package[19] for the R programming environment (http://www.r-project.org). A small offset was added to the intensities before background correction to ensure that there were no negative background-corrected intensities or missing log-ratios and to stabilize the variability of log-ratios in the low-intensity range. Microarray data quality was checked using diagnostic image plots and MA-plots and was found to be satisfactory. Log-ratios were print-tip loess normalised[20], ensuring that low-intensity log-ratios remained of low variability. This made it unnecessary to filter low-intensity spots from the analysis and allowed all spots to be included in the differential expression analysis.

A linear model approach was used to analyse all the microarrays for the two individuals and four cell populations together. Tests of statistical significance between the three time points were conducted for each cell population using empirical Bayes moderated t-tests, which borrow information between genes and give reliable inference even with small sample sizes[21]. The statistical analysis took account of both biological and technical variation. The biological effects of the two subjects were modelled using a common-correlation mixed model analysis[22]. Biological variation between the two individuals was found to make a negligible contribution to the total. The linear model included allowance for probe-specific dye-effects, increasing the precision of the statistical tests. The p-values were adjusted for multiple testing, across all genes and all comparisons, using the method of Benjamini and Hochberg[23] to control the expected false discovery rate (q-value) at less than 5%. For each gene and each comparison, the comparison was considered to be statistically significant if the q-value was less than 5% and the fold change was greater than 50%. The linear model included allowance for probe-specific dye-effects, increasing the precision of the statistical tests.

Cluster version 2.11 and TreeView version 1.60 were used to perform and visualise hierarchical cluster analysis by uncentred correlation with average linkage distance measure (both available at http://rana.lbl.gov/EisenSoftware.htm)[24]. GoStat was used to investigate functional gene ontologies (available at http://gostat.wehi.edu.au/)[25]. The study is MIAME compliant.
